# Anatomical Challenges during Pancreaticoduodenectomy for Adenocarcinoma Head of Pancreas in Presence of Intestinal Rotation Abnormalities: A Report of Two Cases

**DOI:** 10.1055/s-0041-1736670

**Published:** 2021-12-15

**Authors:** Gunjan S. Desai, Sandip Singh, Prasad M. Pande, Prasad K. Wagle

**Affiliations:** 1 Department of Surgical Oncology, S. L. Raheja (A Fortis Associate) Hospital, Mahim (West), Mumbai, Maharashtra, India; 2 Department of General Surgery, Lilavati Hospital and Research Centre, Bandra (West), Mumbai, Maharashtra, India; 3 Department of Surgical Oncology, S. L. Raheja (A Fortis Associate) Hospital, Mahim (West), Mumbai, Maharashtra, India; 4 Department of Surgical Gastroenterology, Lilavati Hospital and Research Centre, Bandra West, Mumbai, Maharashtra, India

**Keywords:** Whipple's procedure, incomplete intestinal rotation, pancreatic cancer, intestinal nonrotation

## Abstract

**Purpose** Pancreaticoduodenenctomy is a complex surgery and the sequence of steps is affected by anatomical variations involving small intestine and major vascular structures. This article depicts our approach to two such cases and highlights the importance of identifying these variations preoperatively on imaging, so as to modify the surgery plan accordingly.

**Cases** We report following two cases of pancreatic head adenocarcinoma (1) one with incomplete intestinal rotation with a replaced right hepatic artery and (2) one with intestinal nonrotation. In both cases, the small bowel was aggregated on the right side of the abdomen, making duodenal mobilization challenging. The surgical approach was modified to prevent injury to these vessels. A superior mesenteric artery (SMA)-first approach helped in early isolation of vascular structures especially when vascular anomaly was also present. Interbowel adhesiolysis, limited kocherisation, tracing all vessels to its origin before division, paracolic anastomotic limb after a longer jejunal limb resection in nonrotation cases, and modification in retropancreatic tunnel creation are few of the key surgical adaptations.

**Conclusion** Asymptomatic Intestinal malrotation is rare in adults and must be identified on preoperative imaging. Resultant intestinal and vascular anatomical variations need meticulous surgical planning and modification of conventional surgical approach for safe performance of PD.

Pancreaticoduodenectomy (PD) is the only curative option for the few patients of pancreatic cancer who present at a resectable stage. However, it is a complex surgery with a high morbidity rate. 1 Presence of anatomical variations, such as intestinal malrotation, which by itself is very rare in adults and replaced right hepatic artery (rRHA) can increase the difficulty of this already challenging procedure. This situation has not been routinely discussed in medical literature owing to its rarity. 2 3

We report two cases encountered in our department with pancreatic head adenocarcinoma (PDAC) who underwent PD for a PDAC in presence of intestinal rotational abnormalities, one of whom also had an rRHA. We emphasize the importance of preoperative imaging to identify these anatomical variations in intestinal rotation that can affect this complex surgery and the measures taken during surgery to ensure a safe pancreaticoduodenectomy in presence of intestinal malrotation.

## Case Reports

### Case 1

A 65-year-old gentleman with no comorbidities presented with progressive jaundice, anorexia, and weight loss since 4 to 5 weeks. He had no similar complaints in past. His clinical examination apart from icterus was normal. Investigations revealed a direct hyperbilirubinemia of 3.3 mg/dL. Ultrasound revealed a hypoechoic lesion in head of pancreas 3 cm × 3 cm in size with dilated common bile duct and intrahepatic biliary radical dilatation.

**Fig. 1:**
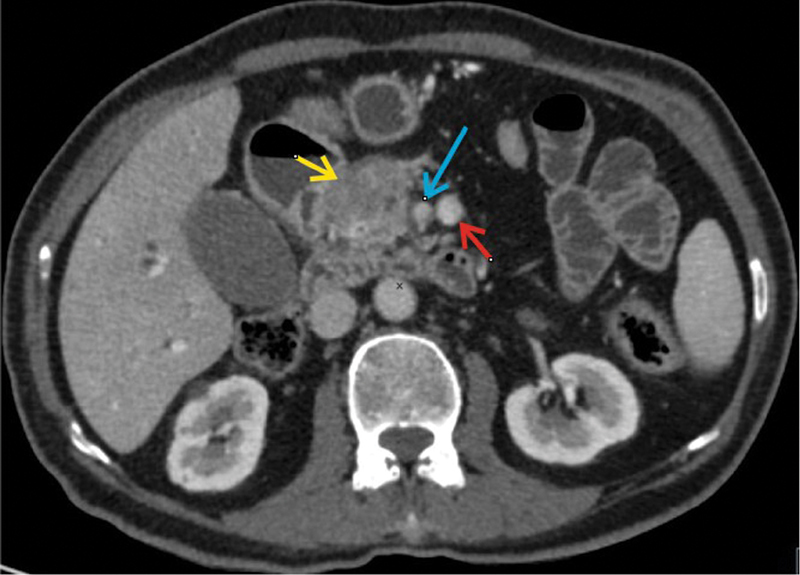
Axial contrast enhanced computed tomography scan image showing mass in the head of pancreas (yellow arrow) and its relation with superior mesenteric artery (blue arrow) and superior mesenteric vein (red arrow).

**Fig. 2:**
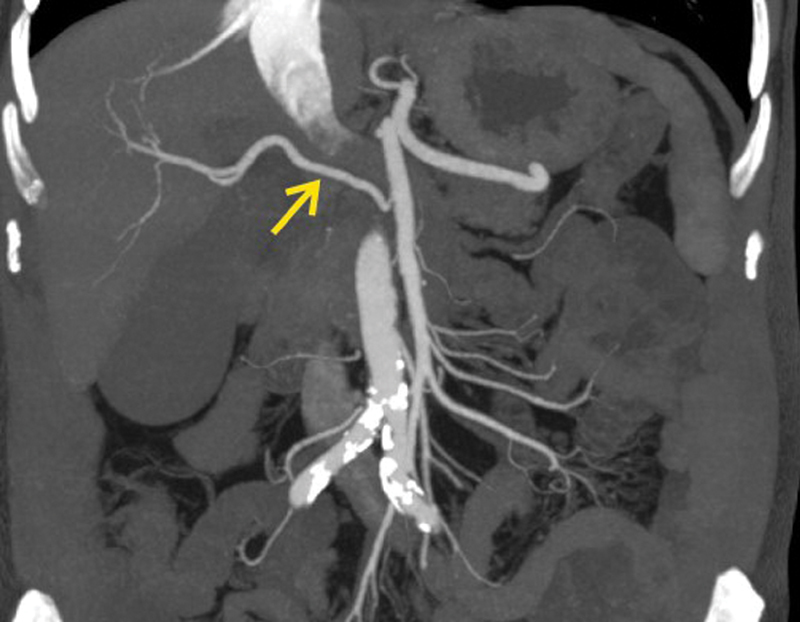
Coronal contrast-enhanced computed tomography scan arterial phase image showing replaced right hepatic artery (yellow arrow) arising from superior mesenteric artery.

**Fig. 3:**
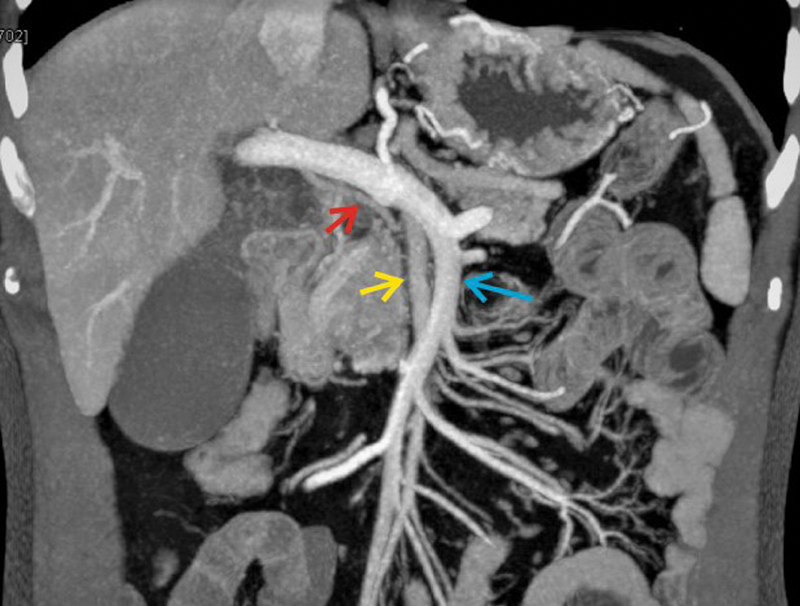
Coronal contrast-enhanced computed tomography scan portal venous phase with multiplanar reformation image showing the reversal of relation between superior mesenteric vein (blue arrow) and superior mesenteric artery (yellow arrow). The red arrow shows the replaced right hepatic artery.

**Fig. 4:**
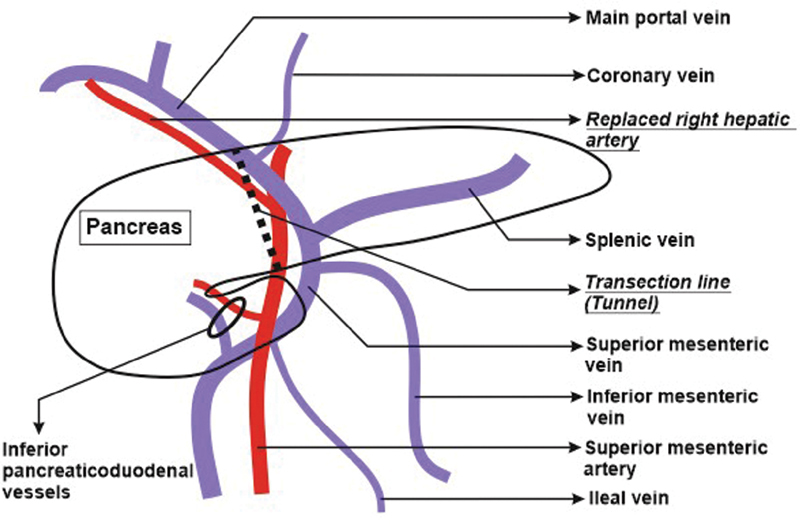
Schematic diagram showing the vascular anatomy in our patient with superior mesenteric artery to the right of superior mesenteric vein. The plane for the retropancreatic tunnel is clearly showing the portal vein at superior end and superior mesenteric artery at the lower limit of the plane.

A pancreatic protocol contrast-enhanced computed tomography (CECT) scan was performed which confirmed the ultrasound findings as shown in Fig. 1 . In addition to this, it showed a replaced right hepatic artery arising from superior mesenteric artery (SMA) as shown in Fig. 2 , and a reversal of relationship of superior mesenteric vein (SMV) and SMA, that is, the artery to the right of vein was seen as shown in Fig. 3 . The complete vascular anatomy is schematically shown in Fig. 4 . The duodenojejunal flexure was in midline. There was no significant lymphadenopathy, no liver lesions, and no free fluid. These findings were suggestive of pancreatic head adenocarcinoma with incomplete intestinal rotation and an rRHA arising from SMA. Carbohydrate antigen 19–9 (CA 19–9) was elevated at 196 U/mL (normal: < 37 U/mL) and Carcinoembryonic antigen was normal. A pylorus-preserving pancreaticoduodenectomy was planned for the patient.

**Fig. 5:**
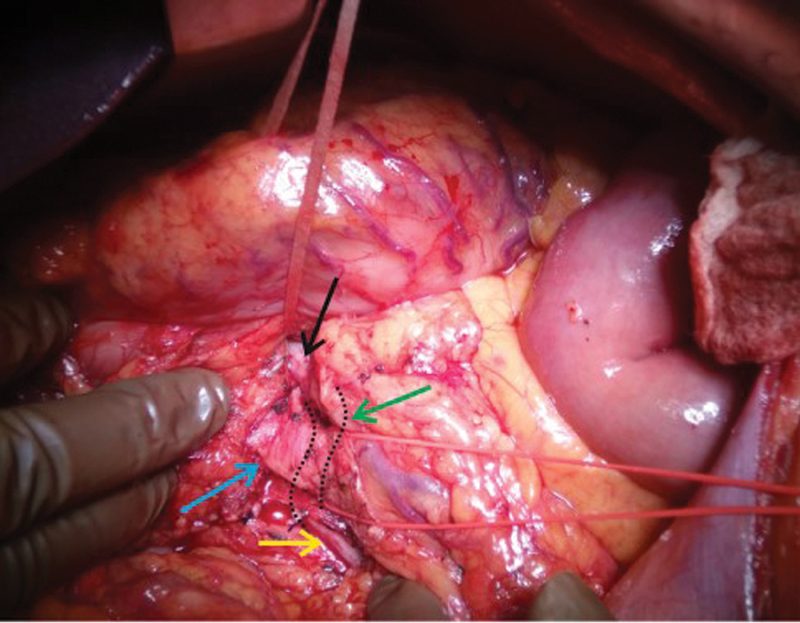
Intraoperative image showing the white loop lifting the pancreas to create the retropancreatic tunnel. The relations of the entire superior mesenteric vein (black, green, and yellow arrow) can be seen to the superior mesenteric artery (blue arrow). The part shown by green arrow was posterior to superior mesenteric artery.

**Fig. 6:**
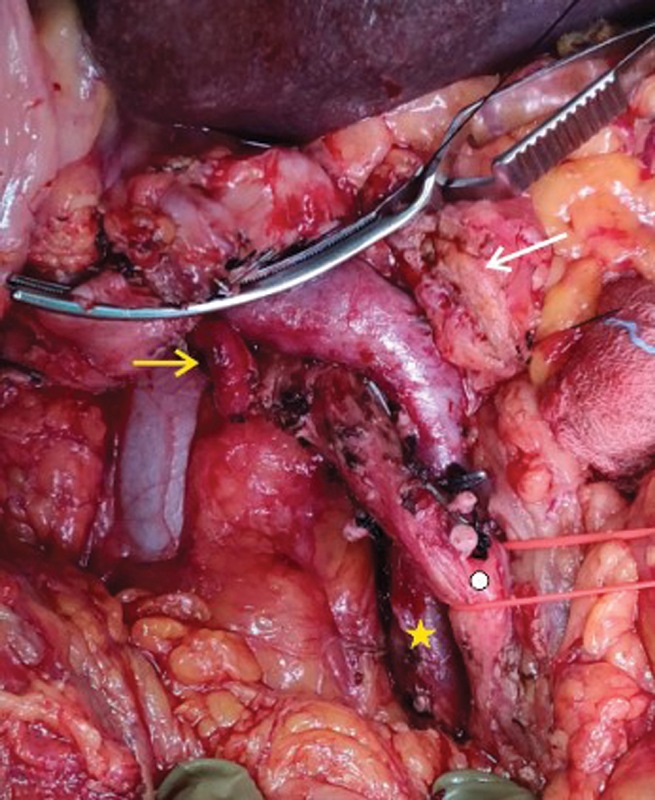
Intraoperative image showing the replaced right hepatic artery (yellow arrow), reverse relation of superior mesenteric vein (star) and superior mesenteric artery (circle). The transacted end of pancreas (white arrow) can also be seen.

During surgery, the small bowel loops were found clumped in right upper abdomen and a Kocher's maneuver was carefully performed after interbowel adhesiolysis to free all the loops till the third part of duodenum. SMA and SMV were then identified and looped at the lower border of pancreas. Hepatoduodenal ligament dissection was then performed to identify the replaced right hepatic artery and the main portal vein. The retropancreatic tunnel was created in a plane above the portal vein superiorly and the SMA inferiorly, and then the plane was widened till the area above SMV ( Fig. 5 ). Uncinate dissection, duodenojejunal flexure mobilization, and standard lymphadenectomy were then performed, followed by division of jejunum, first part of duodenum common bile duct and pancreas. The vascular relations can be seen after resection completion in Fig. 6 .

Reconstruction was performed as the modified Blumgart technique of duct to mucosa pancreatico-jejunostomy with 5–0 polypropylene, single layer interrupted hepatico jejunostomy using 4–0 polypropylene and antecolic stapled posterior gastrojejunostomy. The patient recovered uneventfully and was discharged on the postoperative day 8. Histopathological examination showed moderately differentiated adenocarcinoma confined to pancreas with all margins free and single peripancreatic node out of 18 was positive. The patient is doing well at 4-month follow up on adjuvant 5-fluorouracil based chemotherapy.

### Case 2

A 56-year-old gentleman with no comorbidities presented with progressive jaundice, anorexia, and weight loss of 6 to 8 weeks of duration. He had no similar complaints in past. His clinical examination apart from icterus was unremarkable. Investigations revealed direct hyperbilirubinemia of 10.3 mg/dL. Ultrasound abdomen revealed a hypoechoic lesion in head of pancreas 4 cm × 3 cm in size with dilated common bile duct and intrahepatic biliary radical dilatation.

**Fig. 7:**
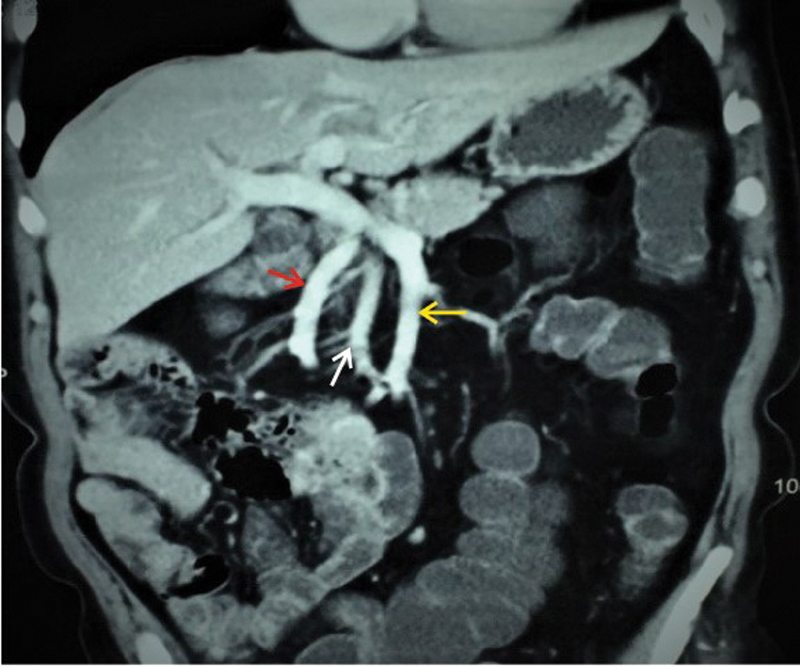
Coronal contrast-enhanced computed tomography scan portal venous phase image showing the ileal (yellow arrow) and jejunal branch (red arrow) of superior mesenteric vein and superior mesenteric artery (white arrow) behind and to the right of superior mesenteric vein.

A pancreatic protocol CECT scan was performed which confirmed the ultrasound findings. In addition to this, there was intestinal nonrotation with entire small bowel on right of abdomen and large bowel on left side. Cecum and hepatic flexure was in midline. SMA was seen coursing between the jejunal and ileal branch of SMV and to right of SMV as shown in Fig. 7 . There was no significant lymphadenopathy, liver lesions, or free fluid. These findings were suggestive of pancreatic head adenocarcinoma with intestinal nonrotation. CA 19–9 was elevated at 237 U/mL (normal: < 37 U/mL) and Carcinoembryonic antigen was normal. A pancreaticoduodenectomy was planned for the patient.

**Fig. 8:**
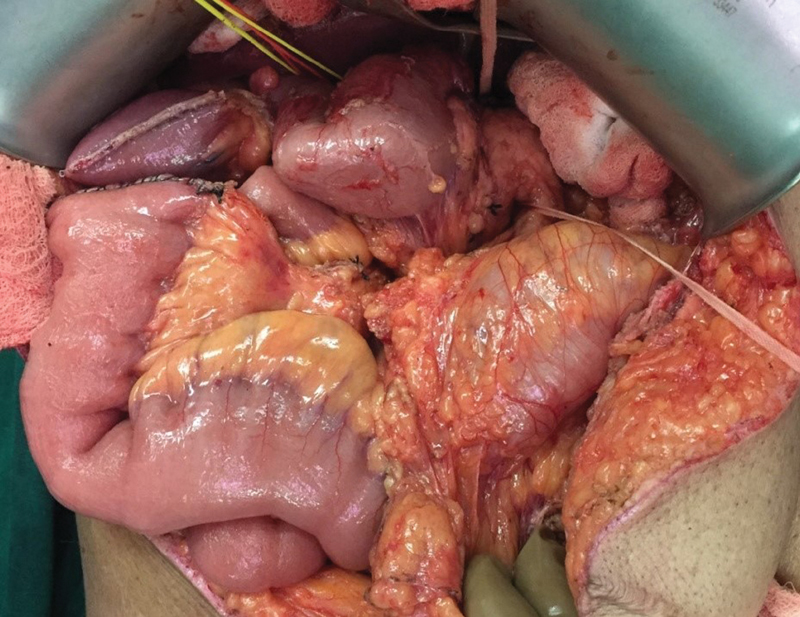
Intraoperative image showing the small bowel loops clumped on right upper abdomen and the cecum and hepatic flexure in midline suggestive of nonrotation.

**Fig. 9:**
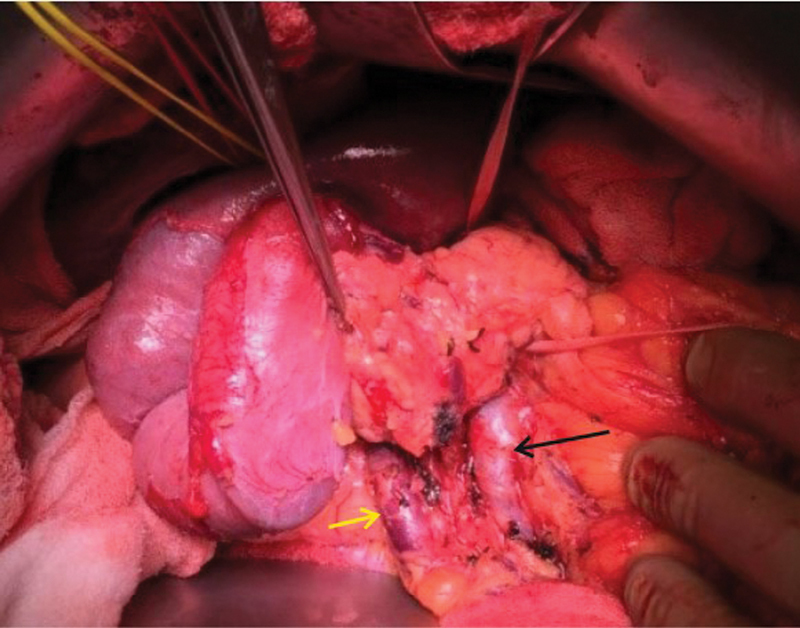
Intraoperative image showing the pancreatic tunnel along the ileal branch of superior mesenteric vein (black arrow) with the superior mesenteric artery coursing posterior to the vein on its right. The jejunal branch (yellow arrow) is to the right of the artery suggestive of nonrotation.

**Fig. 10:**
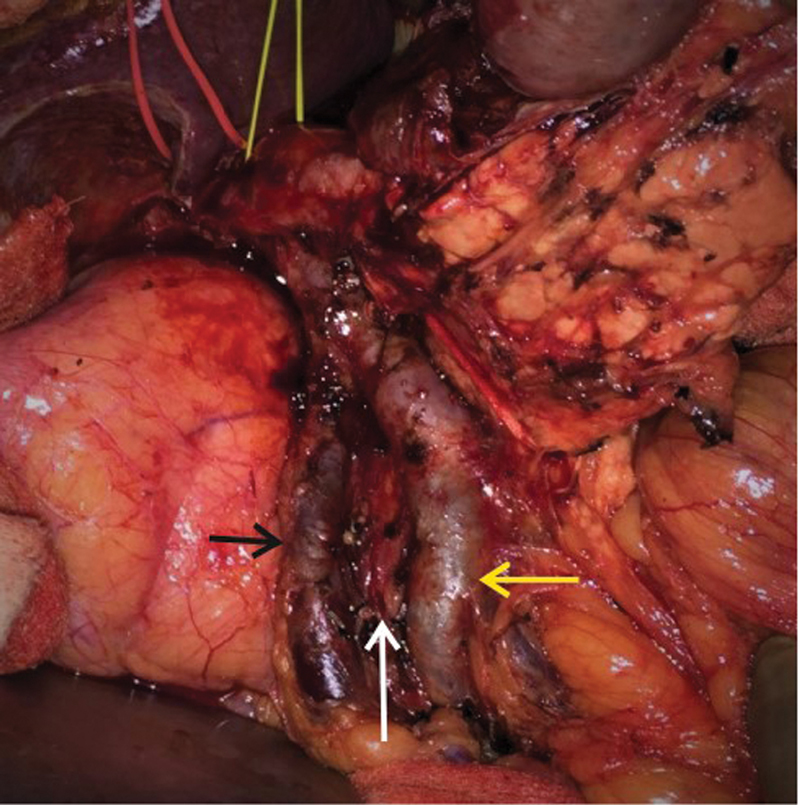
Intraoperative image after specimen resection showing the vascular relations of the superior mesenteric vessels with its ileal branch (yellow arrow), jejunal branch (black arrow), and SMA in between (white arrow).

During surgery, the small bowel loops were clumped in right upper abdomen and the duodenum was vertically linear instead of the usual C loop configuration, with duodenojejunal flexure on the right side. Kocher's maneuver was performed ( Fig. 8 ). The infracolic SMA first approach helps to identify the vascular structures, as well as the replaced hepatic artery from SMA early in surgery, thereby reducing bleeding and it was our approach in this case. SMA and both ileal and jejunal branches of SMV were identified and looped at the lower border of pancreas. Inferior pancreaticodeuodenal vessels were identified to its origin and then divided. Hepatoduodenal ligament dissection was then performed. The retropancreatic tunnel was created in a plane above the portal vein superiorly and the SMV inferiorly, and then the plane was widened till the area above SMA and then above ileal branch of SMV, as we kept dissecting from left to right ( Fig. 9 ). Uncinate dissection, duodenojejunal flexure mobilization, and standard lymphadenectomy were then performed followed by division of jejunum at a slightly longer distance from the duodenojejunal flexure to gain mobility of mesentery to allow length for anastomosis, division of first part of duodenum, common bile duct, and pancreas. The vascular relations can be seen after the resection in Fig. 10 .

Reconstruction was performed by bringing the jejunal loop up from the right paracolic side and not in the conventional retrocolic fashion. The modified Blumgart technique of duct to mucosa pancreaticojejunostomy was performed with 5–0 polypropylene, single layer interrupted hepatico jejunostomy was done using 4–0 polypropylene and stapled posterior gastrojejunostomy was done. The patient had a postoperative chyle leak and superficial surgical site infection which was managed conservatively and he was discharged on the postoperative day 25. Histopathological examination showed tumor confined to pancreas with all margins free and no node out of 16 was positive. The patient is doing well at 3-year follow-up with no adjuvant therapy.

## Discussion

Pancreatic cancers are aggressive cancers and only 20 to 25% can be offered curative treatment in the form of PD as most of them present at an advanced stage. However, PD is a complex surgery with high morbidity rate of 27 to 47%. 1 The significant role of accurate preoperative imaging and surgical planning to help patient selection and reduce morbidity has already been published. 4 Even in the two cases described above, preoperative planning on CECT scan was very important, as it helped us identify the anatomical anomalies present, and thereby plan the two key steps in surgery: (1) the modification of retropancreatic tunnel creation and (2) the modification in orientation of the jejunal loop for a tension free anastomosis.

Embryologically, the four phases of gut rotation around SMA axis include herniation at 5 to 6 weeks of embryonic life, 270° counter-clockwise rotation at 6 to 10 weeks followed by reduction into abdominal cavity, and lastly mesenteric fixation which occurs at 12 weeks. Malrotation encompasses the errors at any of the above four steps which predominantly can be nonrotation, incomplete rotation, reverse rotation, and para-duodenal hernia. This can be for small intestinal preaxial limb, or the ileum + large intestine postaxial limb, or both. Asymptomatic intestinal malrotation is a very rare finding in adults. In a retrospective review, the incidence of intestinal malrotation in adults undergoing hepatobiliary surgery was 3 out of 1,220 cases (0.2%). 3 5

Most of these rotational abnormalities present as duodenal obstruction, cecal volvulus, paraduodenal hernia, and/or intestinal gangrene in the first month of life, and 90% of the cases present in the first year of life. A few recent reports do suggest a higher percentage of presentation at older age. In adults, it can present as recurrent central abdominal pain, diarrhea, malrotation-related pancreatitis due to chronic duodenal obstruction, and volvulus. 6 However, an asymptomatic presentation with incidental detection on imaging as seen in our cases is very rare. 2 3 5 7 A detailed discussion of varied clinical presentations of intestinal malrotation and their management is out of scope of this report. We shall now focus on the implications of the various types of intestinal rotation abnormalities on PD.

The first point to be reemphasized in our cases is that whenever the relation between SMV and SMA is reversed in imaging, there is presence of some form of intestinal rotation abnormality. 8 One pancreatic anatomical variation found to be associated in these cases is a hypoplastic uncinate process. This needs to be remembered and dissection performed accordingly. Heterotaxy syndromes and preduodenal portal vein has also been associated with intestinal rotation abnormalities and should be excluded. 9 Arterial and venous variations are common in presence of intestinal malrotation and these should be looked for. Also, the vascular branches to jejunum arise from the right of SMA rather than the left. These need to be preserved while doing adhesiolysis of the nonrotated or malrotated large bowel from small bowel. 2 5

Mateo et al has described significant vascular variations of celiac trunk, SMV, and SMA in his series of three patients undergoing PD in presence of intestinal malrotation. 3 The vascular variations also affects the resectability of the tumor as has been discussed in one of the published cases where the duodenal carcinoma was operated in spite of arterial involvement, as the involvement was due to an anatomical variation and not due to extensive disease. 4 When in these cases, a replaced right hepatic artery is present or there is variant gastroduodenal or inferior pancreaticoduodenal artery, SMA-first approach is better for quick delineation of the vessels at the inferior border of pancreas and earlier vascular control. 10

The relation of duodenum and cecum to the mesenteric vessels needs to be determined as this will impact the key steps of duodenal mobilization, defining the lower border of pancreas, and forming the anastomotic limb. Abnormal location of ligament of Treitz can make the identification of vascular anatomical landmarks difficult during surgery, especially the mesenteric vein which can lead to intraoperative bleeding complications. These relations need to be studied in detail preoperatively on the imaging studies to avoid such intraoperative difficulties. 2 3 11

In reverse rotation, transverse colon passes posterior to the vessels and duodenum is anterior. In complete nonrotation as in our second case, the mesentery is oriented vertically downward and this will make the mobilization of intestine for anastomosis very difficult. 3 5 Clumping of small bowel in the right abdomen in presence of incomplete intestinal rotation makes Kocher's maneuver difficult. Complete division of Ladd's bands is required in these cases to avoid peritoneal adhesions causing obstruction after surgery while, at the same time, mesenteric vasculature to small bowel needs to be preserved to avoid small bowel ischemia. A longer segment of small bowel needs resection in these cases to create a tension-free anastomosis. Also, the anastomosis will be paracolic and mobilization of hepatic flexure and cecum may be avoided in these cases. 2 3 5 11

Thus, in these cases with intestinal malrotation, the key principles are to perform an accurate preoperative imaging to identify the anomalies present, plan a surgical approach based on the variations present which may be different from the conventional sequence of steps while performing PD, and perform careful adhesiolysis to avoid intestinal devascularization. 5 11 12 A useful technique is to follow each vessel to its origin to identify its variation before division of the vessel to avoid ischemia to critical structures in PD. Jejunal division may need modification to create a tension free anastomosis. 2 3

## Conclusion

PD in presence of intestinal malrotation can be technically challenging and needs careful preoperative planning and intraoperative modifications of conventional steps to avoid complications and achieve a good outcome.
